# A Research on Delayed Thermal Depolarization, Electric Properties, and Stress in (Bi_0.5_Na_0.5_)TiO_3_-Based Ceramic Composites

**DOI:** 10.3390/ma15093180

**Published:** 2022-04-28

**Authors:** Xingru Zhang, Yinan Xiao, Chao Yang, Yuandong Wu, Min Wen, Junke Jiao, Rui Li, Liyuan Sheng, Wenchang Tan

**Affiliations:** 1 Shenzhen Institute, Peking University, Shenzhen 518057, China; zxr_hit@126.com (X.Z.); ynxiao_pkusz@yeah.net (Y.X.); wuy@ier.org.cn (Y.W.); wenm@ier.org.cn (M.W.); twc@pkusz.edu.cn (W.T.); 2 PKU-HKUST ShenZhen-HongKong Institution, Shenzhen 518057, China; lirui19930327@163.com; 3 Key Laboratory for Photonic and Electronic Bandgap Materials Ministry of Education, School of Physics and Electronic Engineering, Harbin Normal University, Harbin 150025, China; yc_hit@outlook.com; 4 School of Mechanical Engineering, Yangzhou University, Yangzhou 225127, China; jiaojunke@yzu.edu.cn

**Keywords:** BNT-based ceramic composite, depolarization behavior, electric properties, stress, ZnO incorporation

## Abstract

Depolarization behavior is one of the main shortcomings of (Bi_0.5_Na_0.5_)TiO_3_-based ceramics. Considering the undesirable efficiency of traditional modification methods, in this paper a series of 0–3 type ceramic composites 0.85(Bi_0.5_Na_0.5_)TiO_3_-0.11(Bi_0.5_K_0.5_)TiO_3_-0.04BaTiO_3_-*x* mol% ZnO (BNKT-BT-*x*ZnO)) were synthesized by introducing ZnO nanoparticles. The results of the X-ray diffraction pattern (XRD) and energy dispersive spectroscopy (EDS) demonstrate that the majority of ZnO nanoparticles grow together to form enrichment regions, and the other Zn^2+^ ions diffuse into the matrix after sintering. With ZnO incorporated, the ferroelectric–ergodic relaxor transition temperature, *T*_F-R,_ and depolarization temperature, *T*_d,_ increase to above 120 °C and 110 °C, respectively. The research on temperature-dependent *P*–*E* loops verifies an attenuated ergodic degree induced by ZnO incorporation. For this reason, piezoelectric properties can be well-maintained below 110 °C. The electron backscatter diffraction (EBSD) was employed to investigate the stress effect. Orientation maps reveal the random orientation of all grains, excluding the impact of texture on depolarization. The local misorientation image shows that more pronounced strain appears near the boundaries, implying stress is more concentrated there. This phenomenon supports the hypothesis that potential stress suppresses depolarization. These results demonstrate that the depolarization behavior is significantly improved by the introduction of ZnO. The composites BNKT-BT-*x*ZnO are promising candidates of lead-free ceramics for practical application in the future.

## 1. Introduction

Ferroelectric ceramics were regarded as an important class of functional materials for the capability of transformation between electric and mechanical energy. After decades of difficult exploration, some excellent species of modified lead-based ceramics, with ideal electromechanical coupling properties, were synthesized and are extensively utilized in electromechanical devices nowadays, such as actuators and sensors [1]. With their commercial market growing briskly, serious environmental issues induced by toxic heavy metal lead gained so much attention that there has been much investigation into lead-free substitutions since the beginning of the 21st century. Under this external demand, the (Bi_0.5_Na_0.5_)TiO_3_ (BNT) ceramic was invented in the 1960s. With high remnant polarization and strong electromechanical coupling effects, BNT-based systems were deemed promising candidates for replacing their lead-based counterparts [2,3]. Having experienced a series of systematic modification, BNT-based ceramics overcame many shortcomings, with the exception of one remaining issue; that is, the deficiency of low operation temperature [4]. Further detailed works reveal that if heated, BNT exhibits the depolarization characteristic, giving rise to domain decomposition [5]. Hence, there is an upper limit of the working temperature, represented by the depolarization temperature *T*_d_. Typically, *T*_d_ of BNT-based ceramics is below 100 °C, which is much lower than their Curie point (often beyond 300 °C) [6]. To achieve their practical application, an elevation of *T*_d_ is needed.

The occurrence of the depolarization phenomenon is related to the characteristic transition from ferroelectric (FE) to ergodic relaxor (ER) state in BNT-based systems [7]. Generally, without electric or mechanical loading, many polar nanoregions (PNRs) reside in the initial BNT ceramic at room temperature. These PNRs possess enough correlation length and lower dynamic activity to cause stronger coupling interaction among them [8]. From the view of statistical physics, their spatially-averaged behavior over the finite region of a matrix is deviated from the temporally-averaged over finite time. This state is usually referred to as nonergodic relaxor (NR) state. With an electric field applied, the PNRs cluster and reorient themselves to irreversibly form domains, inducing the transformation from NR to the ferroelectric state (FE) [9]. Since the activity of PNRs increases during the heating process, the stability of these domains greatly depends on the ambient temperature, bringing on an obvious relaxor phenomenon. BNT-based ceramics turn into the ergodic relaxor state (ER) as long as the threshold, that is, the FE–ER transition temperature *T*_F-R_, is reached [5,7]. Due to domain decomposition, the piezoelectricity of the poled ceramics is lost in ER. Although there may be slight distinctions between *T*_d_ and *T*_F-R_, they both equally originate from the depolarization behavior [10].

Although the underlying mechanism is not understood profoundly, modern ferroelectric theory attributes the depolarization behavior to the inhomogeneous random field [11]. From this point of view, in view of many uncertain factors including the disordered distribution of Bi^3+^ and Na^+^ in A site of perovskite structure, the defects induced by ion volatilization, etc., the local electric field should vary rapidly so that the BNT ceramic behaves as a ferroelectric relaxor with an inadequate *T*_d_. For the same reason, traditional methods of modification, such as doping or forming solid solutions, fail to increase *T*_d_ efficiently, which delays the progress of practical application of BNT-based ceramics. In 2015, by introducing semiconductor ZnO nanoparticles to BNT–BT matrix, Zhang et al. prepared a 0–3 type composite, with no obvious depolarization behavior detected below 120 °C [12], while the *T*_d_ of pure BNT–BT is below 100 °C. To understand the mechanism, a hypothesis that there are bound charges at grain boundaries to compensate for remnant polarization during heating was put forward [12]. In 2016, Mahajan et al. repeated the investigation of the BNT–BT/ZnO composite, and a huge rise in *T*_d,_ of about 40 °C, was observed. Having analyzed the variation of crystal structure, some important impurity phases, such as Bi(Zn_0.5_Ti_0.5_)O_3_, induced by the diffusion of Zn^2+^ ions into the lattice, were suggested as the primary cause of the retarded depolarization behavior, from which a model based on ion diffusion was established [13]. Some related results of BNT–BZT/ZnO composites also support this idea [14].

Considering that the semiconductor nature of ZnO particles is adverse to holding bound charges, the compensation model seems insufficient to explain the increase in *T*_d_ individually. For the ion diffusion model, there is no evidence in published documents that Zn^2+^ doping enhances *T*_d._ Hence, there must be another factor causing the deferred depolarization besides these two effects. Inspired by the fact that external uniaxial mechanical stress is capable of inducing ferroelectric order [11], Riemer et.al focus on the residual thermal stress at boundaries originating from the difference in the thermal expansion coefficient between ZnO and the matrix [15]. A hypothesis of interface-stress suppressing depolarization was put forward. In site temperature-dependent TEM research demonstrates that the regions where depolarization starts to take place is far from the ZnO/matrix interface [16], which is also consistent with the expectation of stress impact. Although the stress model is rational in explaining depolarization, the direct interface stress investigation was not reported. In view of the great significance of increasing *T*_d_, some supplements to this study are necessary. Considering that previous studies on the depolarization behavior of BNT–ZnO composites were mainly carried out for the BNT–BT matrix, which is insufficient to demonstrate the universality of the modification method, in this work a multi-component system of 0.85(Bi_0.5_Na_0.5_)TiO_3_-0.11(Bi_0.5_K_0.5_)TiO_3_-0.04BaTiO_3_ (BNKT-BT), with superior electric properties and proper *T*_d_ [17,18], was selected as the experimental matrix. ZnO nanoparticles were incorporated to introduce boundary stress. The impacts of ZnO on electric properties and depolarization behavior were assessed in detail. In view of the lack of direct stress characterization, stress distribution was measured with the aid of the EBSD for the first time.

## 2. Materials and Methods

Using a traditional solid-state sintering method, the composite ceramic samples 0.85(Bi_0.5_Na_0.5_)TiO_3_-0.11(Bi_0.5_K_0.5_)TiO_3_-0.04BaTiO_3_-*x* mol% ZnO (abbreviated as BNKT-BT- *x*ZnO, *x* = 0, 0.1, 0.2, 0.3) were prepared. To avoid massive diffusion of Zn into the matrix, BNKT–BT were calcined first. The oxides Bi_2_O_3_ (Alfa Aesar 99.9%), TiO_2_ (Acros Organics 99%); and carbonates Na_2_CO_3_ (Acros Organics 99%), K_2_CO_3_ (Acros Organics 99.9%), and BaCO_3_ (Alfa Aesar 99%) were weighed following the stoichiometric formula. Mixed powders were ball-milled in alcohol for 24 h before being pressed. The powder was then calcined at 850 °C for 3 h, to form the principal crystalline phase. Next, ZnO nanoparticles (Aladdin 99.9% 50 nm) were incorporated and ball-milled for 12 h again. After being dried, the powder was blended with PVA (5%), and pressed into pellets under 400 MPa. To reduce element evaporation, these pellets were sintered and buried in the powder of the same component at 1100 °C (*x* = 0), or 1150 °C (*x* = 0.1, 0.2, 0.3) for 1 h. Silver electrode was prepared after that.

The X-ray diffraction pattern of the sample powder was determined using a X-ray diffractometer (Bruker D8 Advance, Hannover, German), utilizing Cu Kα radiation. Micrograph and element distribution was detected using a scanning electron microscope (Gemini SEM 300, Zeiss, Oberkochen, German). The depolarization temperature was measured by a thermally stimulated depolarization current experiment, with a steady heating rate of 3 °C/min, which was described in detail in the literature [8]. The variation of relative permittivity, and loss versus temperature, was determined using a precision LCR meter (Agilent E4980A), ranging from 30 °C to 400 °C. All electric field dependence of polarization was measured using Precision premier II (Radiant Tech. Alpharetta, GA, USA), with a signal frequency of 1 Hz. Poling was processed under an external direct current electric field of 5 kV/mm for 15 min. A Berlincourt meter (Institute of Acoustic, Chinese Academic Society, ZJ-6A, Beijing, China) and an impedance analyzer (Agilent HP4294A) were selected to detect the piezoelectric constant *d*_33,_ and the electromechanical coupling factors *k*_p_ and *k*_t_, respectively. Electron backscatter diffraction (EBSD) was carried out using a field emission scanning electron microscope (TESCAN MIRA3) that was equipped with an EBSD analysis system (Nordlys Max3). The accelerating voltage of the EBSD was 20 kV and step length was 0.06 μm.

## 3. Results and Discussion

**Figure 1:**
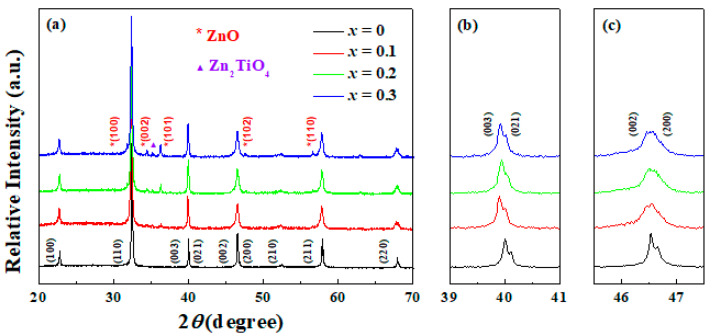
XRD pattern of BNKT-BT-*x*ZnO composites in a 2*θ* range of (**a**) 20° to 70°, (**b**) 39° to 41° and (**c**) 45.5° to 47.5° at room temperature.

The structure of the BNKT-BT-*x*ZnO composites was investigated by means of X-ray diffraction (XRD), and the results are given in Figure 1a. For pure BNKT–BT, there is no secondary phase observed, besides a pattern of a typical perovskite structure within the equipment precision. The major phase belongs to the titanate Bi_0.5_M_0.5_TiO_3_ (M = K, Na), implying that the solid solution BNKT–BT was well-formed. Some important details are depicted in Figure 1b,c. An obvious peak splitting of (003) and (021), located around 40°, indicates a rhombohedral symmetry, while two diffraction peaks (002) and (200), appear around 46.5°, demonstrating a tetragonal symmetry [19]. Hence, pure BNKT–BT ceramic maintains a rhombohedral–tetragonal morphotropic phase boundary (MPB). With ZnO content increasing, another wurtzite diffraction pattern of ZnO appears, suggesting the existence of an independent ZnO phase. Since the interaction of the matrix and ZnO nanoparticles is inevitable, a small quantity of Zn^2+^ ions enters into the matrix lattice during sintering. Considering that Zn^2+^ (0.74 Å) and Ti^4+^ (0.605 Å) are close in radius [13], the substitution of Zn^2+^ for Ti^4+^ occurs easily, shifting the diffraction peaks to a lower angle direction. The maximum value of the shift angle is 0.1°, achieved in the component *x* = 0.3. Moreover, the impurity phase of Zn_2_TiO_4_, the impact of which is often ignored for the non-ferroelectric nature [12], is also detected.

**Figure 2:**
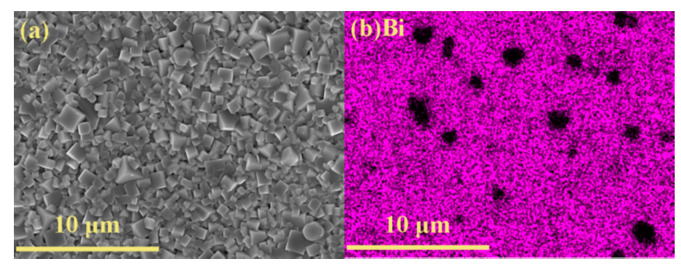

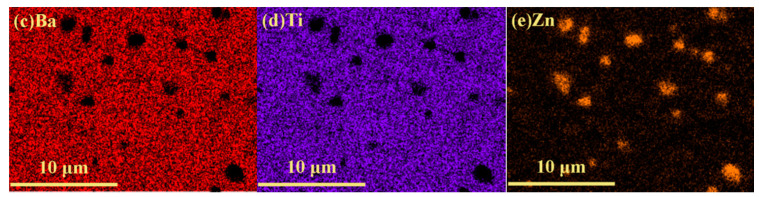
Elemental mapping on the surface of BNKT-BT-0.3ZnO composites, (**a**) morphology of scanning micro area, the distribution of (**b**) Bi, (**c**) Ba, (**d**) Ti, and (**e**) Zn.

The information about Zn distribution was determined by the energy dispersive spectroscopy (EDS), and the element mapping results of BNKT-BT-0.3ZnO are presented in Figure 2. As shown in Figure 2a, under the influence of tetragonal BKT and BT, the grains of the ceramic composites exhibit a cubic shape after sintering. The average grain size reaches 0.91 ± 0.23 μm, demonstrating relatively inadequate growth. According to Figure 2b, Bi locates on a wide-range connected zone, except for some micron-sized regions, and a similar distribution of Ba and Ti is also verified by Figure 2c,d, which suggests the matrix fails to occupy the whole space. On the contrary, as shown in Figure 2e, Zn mainly distributes in these isolated regions. The element mapping results reveal that most ZnO nanoparticles gather to grow into micron-sized enrichment areas, which insert in the matrix to form 0–3 type ceramic composites.

**Figure 3:**
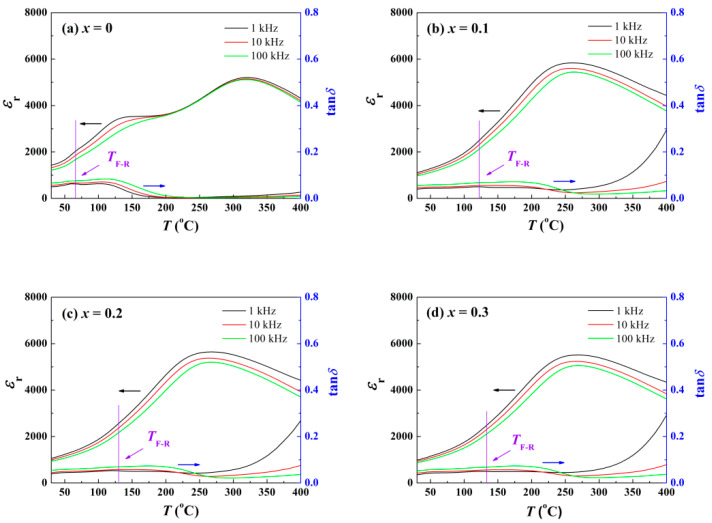
The temperature dependent relative dielectric constant *ε*_r_ and loss spectra tan*δ* of BNKT-BT-*x*ZnO composites: (**a**) *x* = 0, (**b**) *x* = 0.1, (**c**) *x* = 0.2, (**d**) *x* = 0.3.

To inspect the variation in depolarization behavior, the temperature dependence of the relative dielectric constant *ε*_r_ was measured. With temperature increasing, the coupling between electric dipoles decreases, on account of the enhanced dynamic activity of the PNRs. The reorientation of these dipoles better follows the change of high-frequency alternating current (AC) field, giving rise to a frequency-sensitive *ε*_r_. Due to the difference in frequency response of the dipoles in the domains and PNRs, there is a peak in the *ε*_r_-*T* diagram at *T*_F-R_ [20]. With a large number of dynamic PNRs emerging in ER, energy loss enhances, so that another peak appears in the tan*δ*-*T* curves. As shown in Figure 3a, pure BNKT–BT ceramic possess an insufficient *T*_F-R_ of about 65.9 ± 2.7 °C. Since *T*_F-R_ is close to the lower limit of detection, the frequency dependence of *ε*_r_ is readily noticed. When ZnO is incorporated, electric dipoles in the ZnO enrichment areas exhibit their own response to AC loading, and make a contribution to *ε*_r_ distinct from those in the matrix. As a consequence, the *ε*_r_ peak corresponding to *T*_F-R_ becomes obscure. According to Figure 3b–d, *T*_F-R_ acquires a noticeable gain above 60 °C after the introduction of ZnO. The results demonstrate that the incorporation of ZnO inhibits the depolarization process efficiently. The comparison of *T*_F-R_ in the different BNT-based composite ceramics is shown in Table 1.

**Figure 4:**
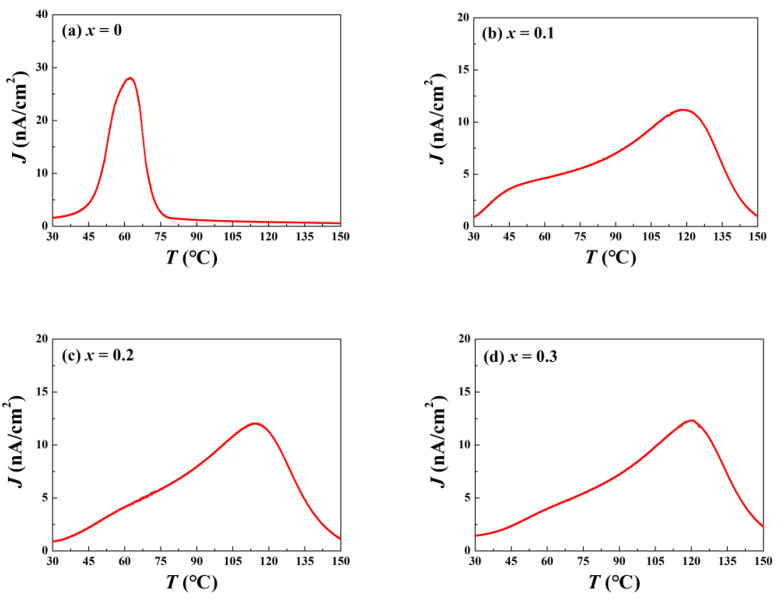
Temperature dependent thermal depolarization current density *J* for BNKT-BT-*x*ZnO composites: (**a**) *x* = 0, (**b**) *x* = 0.1, (**c**) *x* = 0.2, and (**d**) *x* = 0.3.

**Figure 5:**
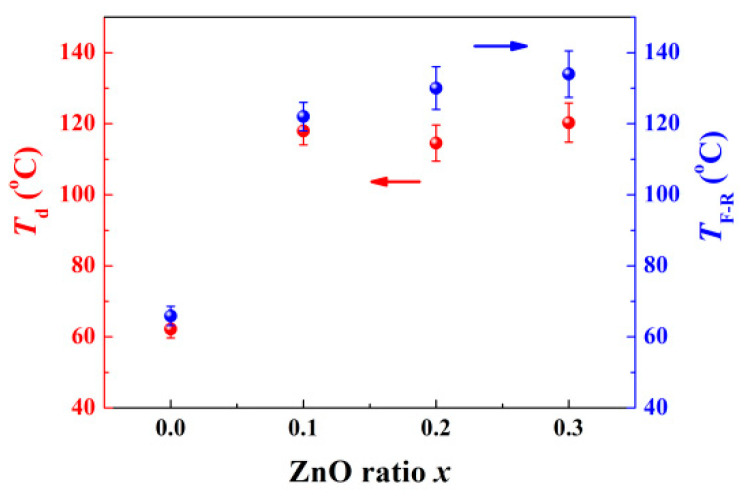
*T*_d_ and *T*_F-R_ as a function of ZnO ratio *x*.

The stability of the domains is a crucial factor for ceramic piezoelectric application. A simple and relevant evaluation for this is the depolarization temperature *T*_d_. Generally, *T*_d_ is estimated using a thermally stimulated depolarization current experiment (TSDC). At low temperatures, the energy provided by thermal fluctuation is insufficient to impact correlation length between PNRs. Although the vibration of positive and negative ions near the equilibrium position is reinforced by increasing temperature, they return to the initial sites after heating, so that the domains remain relatively stable overall [22]. This process produces so-called pyroelectric current in external circuits. However, when the temperature is up to *T*_d_, with a sustained decline in the correlation length of the PNRs, the ion thermal vibration becomes dramatic, and domains decompose into PNRs, which makes the ceramic system enter ER. Once bound charges neutralize inside the ceramics, the free ones attach to the silver electrodes released into the external circuits to yield the depolarization current. Unlike the traditional pyroelectric case, this current, based on domain deterioration, occurs irreversibly [23]. The current density peak, related to the greatest decomposition rate of the domains, corresponds to *T*_d_. The TSDC results are presented in Figure 4. A sole peak, which indicates a continuous one-step depolarization process, is detected for all the components. Figure 4a reveals that the *T*_d_ of the pure BNKT–BT is around 62.2 ± 2.5 °C, which is inferior to many other BNT-based ceramics. However, as long as the ZnO concentration increases to 0.1, an obvious delay for depolarization is observed, and *T*_d_ rises to 118.0 ± 3.9 °C. With greater ZnO content, *T*_d_ increases further. The variation tendency of *T*_d_ and *T*_F-R_ for all the samples is summarized in Figure 5. In view of the limited effect of possible stress, this result does not contradict the stress field model.

**Figure 6:**
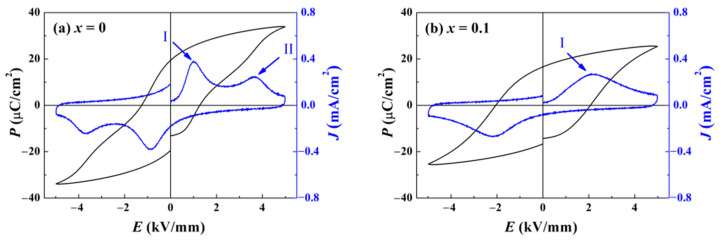

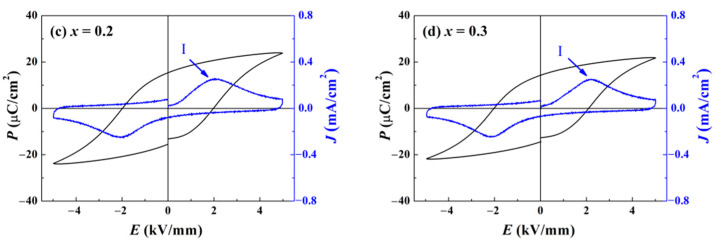
Polarization *P* versus electric field *E* hysteresis curve for BNKT-BT-*x*ZnO composites: (**a**) *x* = 0, (**b**) *x* = 0.1, (**c**) *x* = 0.2, and (**d**) *x* = 0.3 at RT. Peak I denotes a maximum value of the depolarization current density induced by the application of a reverse coercive field. Peak II represents an extreme value of polarization current density caused by domain generation under high electric field.

To inspect the influence of ZnO incorporation on domain reorientation and switching, the polarization *P* versus electric field *E* hysteresis curve (*P*–*E* loop) was measured for all the components at room temperature (RT). As shown in Figure 6a, since the *T*_F-R_ of the component *x* = 0 approaches RT, the *P*–*E* loop exhibits some ergodic characteristics, including a small coercive field *E*_c_ and a shrinking shape. Through calculating the curve of polarization current density *J* versus *E*, two local maximums are discovered. The first (peak I) lies near the position of *E*_c_, for the reason that numerous domains switch dramatically, which implies that peak I arises from the depolarization current [24]. The other (peak II) lies at the electric field that is about three times as great as *E*_c_. This phenomenon suggests a portion of the domains nucleate and develop with the assistance of high loading. Combined with the fact the electric field facilitates the growth of PNRs in relaxor ferroelectrics, the origination of peak II is attributed to the PNRs’ reorientation [25]. With ergodic PNRs transforming into domains, *P* acquire a second boost to form a jagged hysteresis loop. For pure BNKT–BT, the impact of the deep ergodic degree overlays the “hard” doping effect of Zn^2+^, leading to a low *E*_c_. When the ratio of ZnO reaches 0.1, as shown in Figure 6b, the loop shape is remarkably improved as a saturated ferroelectric loop. A maximum only arises around the position of *E*_c_ in the *J*–*E* curve, while peak II vanishes, which demonstrates a decline in ergodic PNRs. An enhanced coercive field *E*_c_ of 2 kV/mm is much higher than that of the component *x* = 0. All these variations confirm that the ergodic degree is greatly decreased. For more ZnO incorporation, as presented in Figure 6c,d, the loop shape barely changes. An evoked drawback is simply ascribed to the decrease in *P*_r_ and *P* maximum, which is attributed to a reduced ratio of the matrix and semi-conductor characteristics of ZnO.

**Figure 7:**
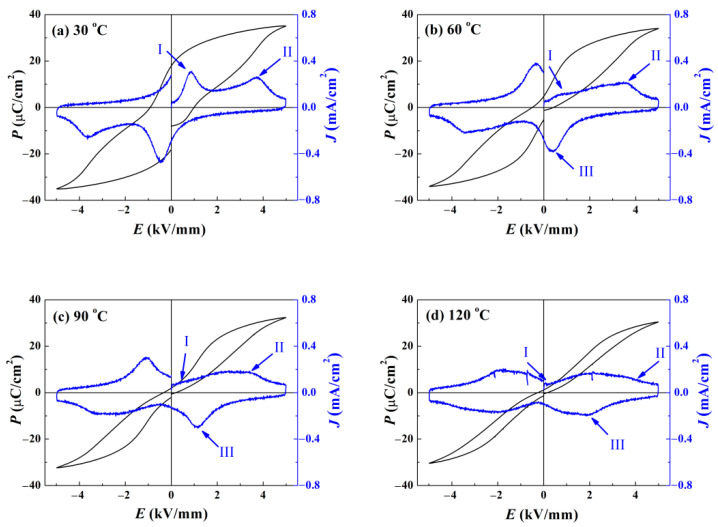
Temperature dependent polarization-electric field *P*–*E* hysteresis curve for the pure BNKT–BT: (**a**) 30 °C, (**b**) 60 °C, (**c**) 90 °C, and (**d**) 120 °C. As temperature increases, Peak I shifts towards the low electric field direction and Peak II shows the opposite movement tendency, which demonstrates an enhanced ergodic degree. The meanings of Peak I and II are the same as those in Figure 6. Peak III appeared above 60 °C denotes the maximum value of depolarization current density originating from the decomposition of the unstable domains.

To reveal the variation of the ergodic degree of the matrix at different temperatures, temperature-dependent *P*–*E* loops were investigated. Figure 7 provides the data obtained for pure BNKT–BT. As depicted in Figure 7a, the shape of the *P*–*E* loop is very similar to that at RT, and only two local maximums arise in the *J*–*E* curve. When the temperature is increased to 60 °C, the waist of the loop becomes slimmer. A small *E*_c_ and *P*_r_ imply a drastically reduced ferroelectricity. With the ambient temperature approaching *T*_F-R_, obvious ergodic relaxor characteristics come into being. Due to the reinforced dynamic activity of ergodic PNRs, a large quantity of domains formed with the aid of high loading decompose into PNRs again when the electric field falls off. As a consequence, a depolarization current density peak III exists in the *J*–*E* curve. For a rising temperature of 90 °C, the loop shape becomes more contracted with a negligible *E*_c_ and *P*_r_. The location of peak III moving in the direction of high electric field suggests that the threshold preventing domains from decomposing is increasing [26]. For a higher temperature of 120 °C, *E*_c_ and *P*_r_ almost vanish, verifying the total loss of piezoelectricity. The maximum of *P* at the electric field of 5 kV/mm starts to drop, due to the stricter condition for inducing domains.

**Figure 8:**
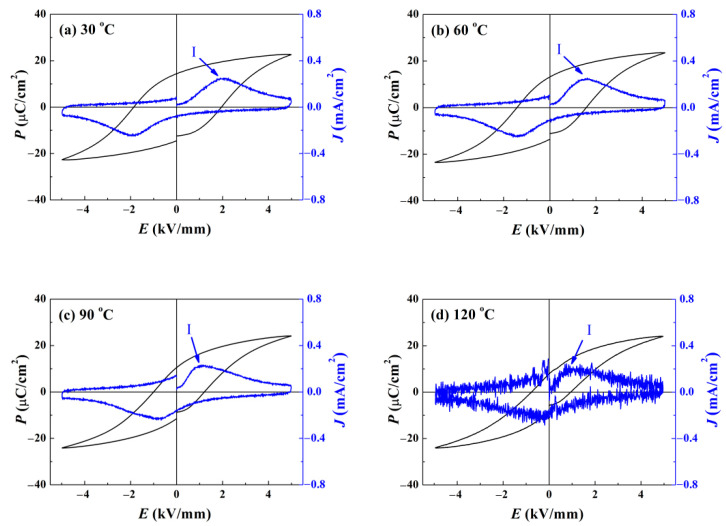
Temperature dependent polarization–electric field *P*–*E* hysteresis curve for the component *x* = 0.3: (**a**) 30 °C, (**b**) 60 °C, (**c**) 90 °C, and (**d**) 120 °C. Compared to pure BNKT-BT ceramic at the same temperature, no other evidences of the notable changes of the ergodic degree were detected except for the shift of Peak I towards the low electric field direction, which demonstrates an improved temperature stability.

For a comparison, the temperature-dependent *P*–*E* loops for the component *x* = 0.3 are presented in Figure 8. A typical saturated ferroelectric hysteresis loop appears at 30 °C, and only one maximum in the corresponding *J*–*E* curve. For the temperatures ranging from 30 °C to 90 °C, peak I slightly shifts towards the low-field direction, for the reason that domains are easier to switch at higher temperatures. No other evidence of the notable changes of the ergodic degree, such as the occurrence of peak II or peak III as in Figure 7, is detected. Hence, it is rational to predict that piezoelectric performance could be well-sustained over this temperature range. Even if the temperature is increased to 120 °C, the *P*–*E* loop still holds its shape relatively well, and the non-zero value of the remnant polarization *P*_r_ hints that piezoelectricity is partially preserved. These results demonstrate that the introduction of ZnO enrichment areas is beneficial to inhibit the elevation of the ergodic degree. For the BNKT-BT-*x*ZnO composite system, the phenomenon that polarization *P* declines with applied electric field *E* increasing does not emerge, which means the conductivity is well suppressed.

**Figure 9:**
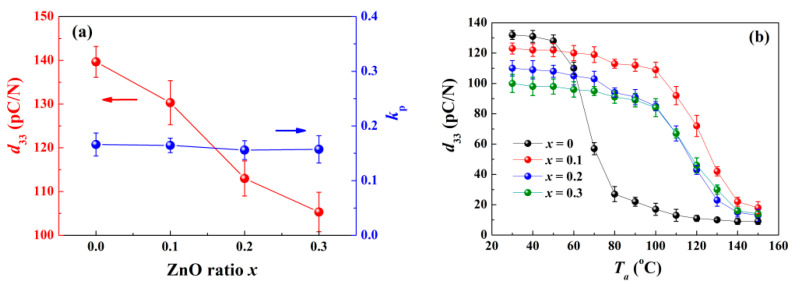
(**a**) Piezoelectric constant *d*_33_ and planar electromechanical coupling factor *k*_p_ as a function of ZnO ratio *x*, (**b**) remaining *d*_33_ as a function of annealing temperature *T*_a_ for all the components.

To evaluate the piezoelectric performance of the samples, two indicative parameters, i.e., the piezoelectric constant *d*_33_ and planar electromechanical coupling factor *k*_p_, are measured in Figure 9a. While the content of ZnO is increasing, both *d*_33_ and *k*_p_ decline because of the reduced relative ratio of the ferroelectric matrix, which means partial loss of piezoelectricity [12]. Figure 9b provides the relationship between the annealing temperature *T*_a_ dependence and the remaining *d*_33_. The remaining *d*_33_ for the pure BNKT–BT ceramic drops rapidly in the temperature range of 60−80 °C. Since the phase transition from FE to ER finishes above 80 °C, only a negligible remaining *d*_33_ is detected. The components with ZnO incorporated show an improved temperature stability, holding the performance at a high level until 110 °C is reached. The following declining phase for the component *x* = 0.3 tends to be smoother. In view of superior properties when compared to the other components, the component *x* = 0.1 is more suitable for real application.

**Figure 10:**
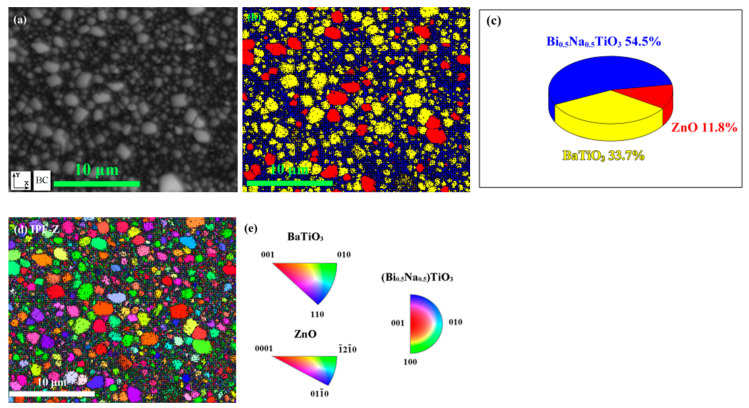
(**a**) Surface morphology of EBSD scanning region in the component *x* = 0.3, (**b**) phase distribution in the selected region, (**c**) percentage of different phases in the scanning region, (**d**) crystal orientation map along Z axis, and (**e**) inverse pole figure (IPF) color key.

The patterns based on the electron backscatter diffraction (EBSD) were employed to understand the distribution of strain. A scanning area in the component *x* = 0.3, shown in Figure 10a, was selected for the purpose. According to the corresponding phase distribution and percentage offered by Figure 10b,c, three types of configurations exist in this area. The local regions, marked in blue and yellow, possess crystal structures close to Bi_0.5_Na_0.5_TiO_3_, and BaTiO_3_, respectively, which represents the distribution of the matrix; while the other regions, marked in red, have structures similar to ZnO, standing for the ZnO enrichment areas diffused in the matrix. The individual orientations of grains along the *Z*-axis of the laboratory coordinate system are provided in Figure 10d. Comparing the colored grains and the inverse pole figure color key in Figure 10e, it is revealed that both the grains of ZnO and the matrix orient themselves randomly. Since the potential stress field does not induce texture, the possibility of texture affecting depolarization behavior is excluded.

**Figure 11:**
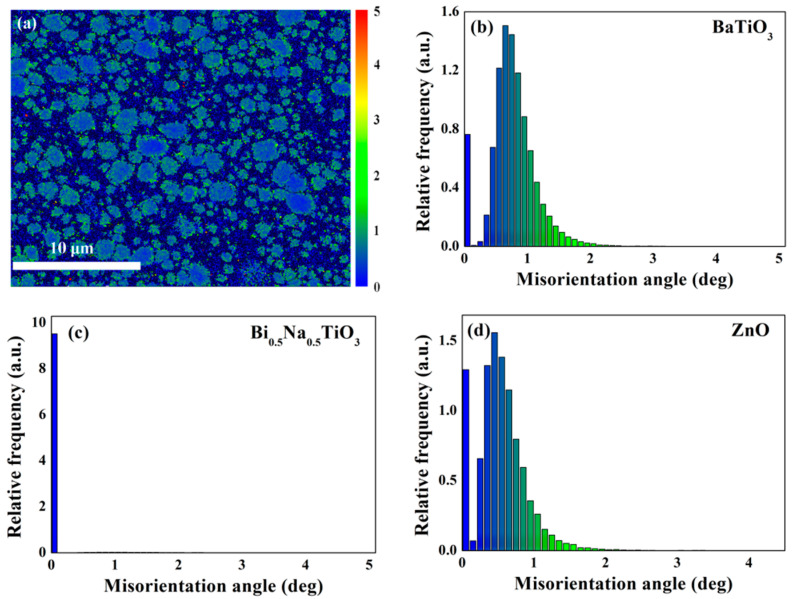
(**a**) Local misorientation distribution image for the selected region, and the relative fraction of sampling sites versus different misorientation angles for (**b**) BaTiO_3_, (**c**) Bi_0.5_Na_0.5_TiO_3_, and (**d**) ZnO.

More pronounced strain occurs in the regions of composites where stress is concentrated. By means of calculating the displacements between adjacent sampling sites in the EBSD pattern, a speculation on stress field distribution is built up [27,28]. For the purpose, Figure 11 provides the image of local misorientation and the relative fraction of sampling sites corresponding to different misorientation angles. According to the Figure 11b–d, the maximum of the misorientation angle approaches 4.5°, which corresponds to the tiny areas marked in red in Figure 11a. All these areas are immersed in the blue region of the Bi_0.5_Na_0.5_TiO_3_ matrix. In view of the effect of abundant grain boundaries on measurement accuracy, this portion of misorientation can, in fact, be ignored. The majority of sampling sites have misorientation angles in intervals of 0° to 1.5°, which corresponds to the color transition region from blue to green. As shown in Figure 11a, grain boundaries are prone to exhibiting green, while the inner parts of grains are blue. Since the green area means larger strain, it is confirmed that the stress field prefers to emerge near the boundaries. With the stress effect of pinning domains, the hypothesis that stress field hinders domain decomposition to postpone depolarization is reasonable [29]. In addition, no particular stress effect is detected around the boundaries of BNKT-BT/ZnO. The result does not support the previous prediction, based on the difference in the thermal expansion coefficient of ZnO and the matrix.

## 4. Conclusions

In this paper, 0–3 type ceramic composites BNKT-BT-*x*ZnO were synthesized using a traditional solid-state sintering method. The results of XRD and EDS demonstrate that the majority of ZnO nanoparticles grow together to form enrichment regions, and the other Zn^2+^ ions diffuse into the matrix to substitute Ti^4+^ in B sites. After the introduction of ZnO, the variation of temperature-dependent *ε*_r_ demonstrates a *T*_F-R_ increase to above 120 °C, while the TSDC confirms *T*_d_ reaches above 110 °C. The research on *P*–*E* loops at various temperatures verifies an attenuated ergodic degree induced by ZnO incorporation, which ensures a greater remnant polarization at the same temperature. The measurement of the remaining *d*_33_ shows that piezoelectric properties are well-maintained below 110 °C. All these results demonstrate that the depolarization phenomenon of composites is improved greatly. The EBSD orientation maps reveal the random orientation of all grains, excluding the impact of texture on depolarization. The local misorientation near the boundaries is more pronounced than in the other parts, implying stress is more concentrated there. Considering the potential pinning effect, the stress model for postponed depolarization is supported. The composites BNKT-BT-*x*ZnO are promising candidates of lead-free ceramics for real application in the future.

## Figures and Tables

**Table 1:** The comparison of *T*_F-R_ in different BNT-based composite ceramics.

BNT-Based Composite Ceramics	*T*_F-R_ (°C)	The Increment of *T*_F-R_ Relative to the Matrix (°C)	Reference
BNKT-BT-0.3ZnO	133.6	67.8	This work
0.94BNT-0.06BT-0.3ZnO	125	27	[12]
0.94BNT-0.06BT-0.3ZnO	140	40	[13]
0.93BNT-0.07BT-0.3ZnO	114	25	[21]
0.8BNT-0.2BKT-0.15Al_2_O_3_	227	111	[4]

## Data Availability

Data sharing not applicable.
